# Preservation of Xinyu Tangerines with an Edible Coating Using *Ficus hirta* Vahl. Fruits Extract-Incorporated Chitosan

**DOI:** 10.3390/biom9020046

**Published:** 2019-01-28

**Authors:** Chuying Chen, Zhengpeng Nie, Chunpeng Wan, Jinyin Chen

**Affiliations:** 1 Jiangxi Key Laboratory for Postharvest Technology and Nondestructive Testing of Fruits & Vegetables, Collaborative Innovation Center of Postharvest Key Technology and Quality Safety of Fruits and Vegetables, Jiangxi Agricultural University, Nanchang 330045, China; cy.chen@jxau.edu.cn (C.C.); nzp2018@126.com (Z.N.); 2 Pingxiang University, Pingxiang 337055, China

**Keywords:** edible coating, Xinyu tangerines, *Ficus hirta* Vahl. fruits, chitosan, preservation effect

## Abstract

Xinyu tangerine is a citrus fruit that has enjoyed great popularity in China for its fewer dregs and abundant nutrients. However, it is considered an easily perishable fruit that is vulnerable to various pathogenic fungal infections, especially by *Penicillium italicum*, which reduces its storage life and commercial value. Normally, to reduce the losses caused by fungal deterioration of harvested fruit, polysaccharide-based edible coating, containing natural antimicrobial agents (e.g., plant extracts), have been applied. In current study, we evaluated the effects of *Ficus hirta* Vahl. fruits extract (FFE)–incorporated chitosan (CS) edible coating on Xinyu tangerines during cold storage at 5 °C. The results showed FFE has efficacy as an antifungal against *P. italicum* in a dose-dependent manner *in vivo*, with an EC_50_ value of 12.543 mg·mL^−1^. It was found that the edible coating of FFE–CS exhibited a higher reduction of total soluble solid (TSS), titrable acid (TA), and ascorbic acid (AsA) content by reducing the fruit decay rate, weight loss, respiration rate, and malondialdehyde (MDA) content during cold storage at 5 °C. Moreover, the activities of protective enzyme such as superoxide dismutase (SOD), peroxidase (POD), and phenylalanine ammonia-lyase (PAL), which have been linked with reactive oxygen species (ROS) and the phenylpropanoid pathway, were higher in the FFE–CS-coated fruits. On the basis of these study results, the FFE–CS edible coating could reduce postharvest loss and enhance the storability of Xinyu tangerines due to the *in vivo* antifungal activity of FFE.

## 1. Introduction

Xinyu tangerines (*Citrus reticulata* Blanco) cv. Pengjia no. 39, a local mandarin bred from ‘běn dì zǎo’ (*Citrus reticulata* Blanco) cv. Succosa in Huangyan City (Zhejiang Province, China), enjoy great popularity in China for their delicious taste, abundant nutrients, uniform color, fewer dregs, and delicate pulp. However, the fruits are highly susceptible to fungal pathogen infection and mechanical injury during storage due to their rich nutritional content and tender peel. Moreover, the harvested Xinyu tangerines have a high respiration rate and water loss, and are easily attacked by pathogens at room temperature [1,2]. For these reasons, Xinyu tangerines usually have short shelf-life and quick deterioration of nutrients, which seriously reduces their storability and postharvest fruit quality. Therefore, it is necessary to develop effective preservation strategies for this important fruit crop.

Till now, several preservation strategies, including cold storage, hot water, gamma irradiation, and edible coating have been applied for the postharvest preservation of Xinyu tangerines [3,4,5]. The edible coating has attracted much attention due to its potential to carry natural antimicrobial agents (such as plant extracts, essential oils, and active antimicrobial ingredients that reduce the risk of pathogen growth on fruits and vegetables), as well as for its easy accessibility and eco-friendly nature [6,7,8]. Edible coatings composed of polysaccharides, including alginate, celluloses, chitosan, and starch, have successfully been applied for harvested fruits. Chitosan (CS) is a natural biodegradable polysaccharide derived from the deacetylation of chitin, and has been used as an effective edible coating to suppress the respiration and water transpiration of fruits and vegetables during storage [7,9,10]. However, the effect of CS coating for the preservation of Xinyu tangerines is not satisfactory, likely due to its low solubility and film-forming ability and its insufficient properties as a mechanical antioxidant and antimicrobial agent.

*Ficus hirta* Vahl. is well-recognized plant for its five-fingered leaf shape and mature fruits resembling wild peach. *Ficus hirta* (family Moraceae) is widely distributed in Southern China and is used for the treatment of constipation, inflammation, postpartum hypogalactia, and tumors and cancers [11,12,13,14]. Previous studies have reported the antimicrobial activity of the roots and fruits of *F. hirta* against *Escherichia coli*, *Staphylococcus aureus*, *Alternaria citri*, *Botrytis cinereal*, and many others *in vitro* [15,16]. Moreover, the crude aqueous, ethyl acetate, and butanol extracts of Wuzhimaotao exhibited cytotoxic effects on HeLa cells [14]. The fruit of *F. hirta* Vahl. is a famous herbal medicine in Southern China, named ‘Wú Zhǐ Máo Táo Guǒ’ in Chinese pharmacopoeia, and a traditional plant resource used both as medicine and food by Hakka people [10,17,18]. Wan and colleagues [19] have reported that pinocembrin-7-O-β-D-glucoside, an important flavonoid isolated from the ethanolic extracts of *F. hirta* fruits, has potent antifungal effects against *Penicillium italicum* in citrus fruit. However, hardly any published reports exist on the effects of *Ficus hirta* Vahl. fruits extract (FFE)–incorporated chitosan (CS) FFE–CS coating for Xinyu tangerine preservation. Thus, the aims of the current study were to investigate the *in vivo* antifungal efficacy of FFE for controlling blue mold caused by *P. italicum* in citrus fruit, and to evaluate the preservation effect of FFE incorporated into CS-based edible coatings on harvested Xinyu tangerines during cold storage.

## 2. Materials and Methods 

### 2.1. Materials

Xinyu tangerines (*Citrus reticulata* Blanco) cv. Pengjia No. 39 used throughout this study were harvested from Mahong Garden-Spot, located in the Yushui district of Xinyu City (Jiangxi, China), during late October 2016. The fruits were selected on the basis of health, consistent size (72–85 g), uniform color (2.5–3.2), and features of commercial maturity (i.e., free of mechanical injury, blemish, and disease). 

### 2.2. Fungal Pathogen and Medium

*P. italicum* was isolated from infected citrus fruits showing the typical symptoms of blue mold in Jiangxi Key Laboratory for Postharvest Technology and Nondestructive Testing of Fruits & Vegetables (Nanchang, Jiangxi Province, China) and identified by Miaolian Xiang (College of Agricultural, Jiangxi Agricultural University, Nanchang, China). Potato dextrose agar (PDA: 200 g peeled potatoes, 20 g glucose, 18 g agar powder, and 1 L distilled water) medium was used for the maintenance of *P. italicum*, and the pure culture was sustained at 25 °C for 7 days.

### 2.3. Extraction of FFE

The fruits of *F. hirta* Vahl. (origin: Meizhou, Guangdong Province, China) were purchased from Huafeng Pharmacy in Zhangshu city (Jiangxi Province, China) and authenticated by Shouran Zhou (College of Basic Medicine, Jiangxi University of Traditional Chinese Medicine, Nanchang, China). The dried fruits were powdered in a FW-100 grinder (20 mesh, Taisete Instrument Inc., Tianjin, China) after drying below 40 °C for 15 h. The FFE was obtained using an ultrasonic-assisted method described by Chen et al [16]. The dry FFE was kept at −20 °C and reconstituted with distilled water to give the desired concentration of 20 mg/mL (dry extract / distilled water, *w*/*v*) for further use. 

### 2.4. Antifungal Efficacy of FFE on In Vivo Mycelial Growth of P. italicum

The selected Xinyu tangerines were washed with 0.5% sodium hypochlorite for 2 min, rinsed with distilled water, and air-dried on a benchtop before wounding [2]. One wound (4 mm wide and 2 mm deep) was made per fruit on the equatorial side using a sterile needle. 15 μL of FFE at 20, 10, 5, and 2.5 mg/mL was pipetted into the wounds. Controls were injected with the same volume of sterile distilled water substituted for FFE. After 60 min, the wounds of FFE-treated and control fruits were reinjected with 15 μL of suspension of *P. italicum* spore (10^6^ CFU/mL). There were three replicate trials of fifteen fruits per treatment with completely random allocation, and all experiments were performed twice. The lesion diameters of the fungal colonies were recorded in millimeters after 7 days of incubation at 25 °C. The control efficacy was expressed in terms of percentage of mycelia growth inhibition (MGI) and calculated using Equation (1):

(1) $$\mathrm{MGI}\;\left(\%\right)=\frac{\mathrm{Dc}-\mathrm{Dt}}{\mathrm{Dc}}\times \;100,$$

where Dc and Dt were the averages of lesion diameters (mm) in the control and in the treatment, respectively.

### 2.5. Preparation of FFE–CS and 2.0% CS Coatings

2.0% (*w*/*v*) CS solution was prepared by dissolving 10.0 g of chitosan (Sinopharm Chemical Reagent Co., Ltd., Shanghai, China) in 400 mL of 0.5% (*v*/*v*) acetic acid solution. Dry FFE (6.25 g) was dissolved in moderate distilled water and put into the above CS coating. After agitation for 60 min, the pH of the solution was adjusted to pH 5.4 using 1.0 M NaOH, and the total volume of FFE–CS coating was made up to 500 mL. The 2.0% CS coating was prepared in the same way without addition of FFE. 

### 2.6. Coating Treatments

The selected Xinyu tangerines were washed with tap water and air-dried at room temperature for 4 h, then coated by dipping in 2.0% CS coating and/or FFE (FFE–CS and 2.0% CS coating, respectively) for 1 min, while the control group was dipped in 0.5% acetic acid solution (pH 5.4). After drying, the coated and control fruits were individually film (16 cm × 12 cm, Lingqu fresh packaging products Co. Ltd., Guilin, China)-packaged. After being pre-cooled at 10–12 °C for 12 h, all fruits were stored at 5 ± 0.5 °C, and 85–90% relative humidity (RH), for 90 days. 

### 2.7. Physicochemical Measurements of Xinyu Tangerines 

#### 2.7.1. Total Soluble Solid (TSS) and Titrable Acid (TA)

The TSS and TA contents of juice extracted from 10 fruits from the coated and control groups was measured using a RA-250WE Brix-meter (Atago, Tokyo, Japan) and PAL-ACID1 digital acidimeter (Atago), and the results were expressed as a percentage. 

#### 2.7.2. Ascorbic Acid (AsA)

AsA content in the extracted juice was determined by titration with 2,6-dichlorophenol indophenol and expressed in mg per 100 g juice (mg/100 g) 

#### 2.7.3. Decay Rate

Decay rate was visually evaluated using the same 120 fruits per treatment per replicate, and expressed as the percentage of rotted fruits. For this test, 360 fruits from each treatment were used and evaluated on days 0, 15, 30, 45, 60, 75, and 90.

#### 2.7.4. Weight Loss

Weight loss was determined by single fruit weighting, using Equation (2). For this test, 15 fruits were used and evaluated on days 0, 15, 30, 45, 60, 75, and 90.

Weight loss (%) = (*W_i_ – W_0_*)*/W_0_ ×* 100, (2)

where *W_0_* and *W_i_* are the initial and final weight (g), respectively, of the same fruits.

#### 2.7.5. Respiration Rate

The respiration rate was determined based on a method described by our previous study [2]. Respiration rate was measured by CO_2_ production, using Equation (3), and expressed as mg·kg^−1^·h^−1^.

(3) $$\mathrm{Respiration}\;\mathrm{rate}\;\left({\mathrm{mg}\;\mathrm{kg}}^{-1}h^{-1}\right)=\frac{\Delta {\mathrm{CO}}_{2}}{100}\times V_{\mathrm{headspace}}\times \frac{1000}{m}\times \frac{60}{t},$$

where *∆CO_2_* is the increment of CO_2_ concentration, *V_headspace_* is the empty volume of the container (mL), *m* is the mass of Xinyu tangerines (g), and *t* is recording time (min), respectively.

#### 2.7.6. Malondialdehyde (MDA)

MDA content was measured according to the method described by Hodges and coworkers [20]. Pericarp tissues of 10 fruits were ground in a MM 400 frozen grinder (Retsch GmbH., Arzberg, Germany), and 2.0 g of powder was homogenized in 25 mL of ice-cold 50 mM phosphate buffer (pH 7.8) containing 1 mM ethylenediaminetetraacetic acid (EDTA) and 2% (*w/v*) polyvinylpyrrolidone (PVP), and centrifuged at 12,000 × g for 20 min at 4 °C. 2 mL of the collected supernatant was mixed with 2 mL of 0.5% (*w*/*v*) thiobarbituricacid (TBA), and further incubated in boiling water for 30 min. After being cooled and centrifuged at 6000 × g (5804R, Eppendorf) for 10 min, the absorbance of supernatant was measured at three different wavelengths (450, 532, and 600 nm) using an M5 microplate reader (Molecular Devices Corporation, Sunnyvale, California, USA). The MDA content was calculated according to Equation (4) and expressed as mmol·g^−1^ FW.

MDA content (mmol·g^−1^) = 6.452 × (*A*_532_ − *A*_600_) − 0.559 × *A*_450_. (4)

#### 2.7.7. Protective Enzyme Activities

Aliquots of fruit peel powder (2.0 g) were homogenized with various ice-cold extraction buffers to prepare extracts for assay of the following protective enzymes: 10 mL of 50 mM ice-cold phosphate buffer (pH 7.8) containing 1 mM EDTA, 5 mM DTT, and 2% (*w*/*v*) PVP for superoxide dismutase (SOD, EC 1.15.1.1); 8 mL of 100 mM ice-cold acetate buffer (pH 5.5) containing 1 mM polyethylene glycol (PEG), 4% (*w*/*v*) PVP, and 1% (*w*/*v*) Triton X-100 for peroxidase (POD, EC 1.11.1.7) and polyphenol oxidase (PPO, EC 1.10.3.1); 5 mL of 50 mM ice-cold Tris-HCl buffer (pH 8.8) containing 15 mM β-mercaptoethanol, 5 mM AsA, 5 mM EDTA, 1 mM phenylmethylsulfonyl fluoride (PMSF), and 0.15% (*w*/*v*) PVP for phenylalanine ammonia-lyase (PAL, EC 4.3.1.5). All homogenates were centrifuged at 12,000 × g (5804R, Eppendorf) for 30 min at 4 °C. The supernatants were then collected and used for the enzyme activity assays.

SOD activity was assayed by measuring its ability to inhibit the photoreduction of nitroblue tetrazolium (NBT) according to the method of Sala and Lafuente, with slight modifications [21]. The reaction mixture consisted of 1.5 mL PBS (50 mM), 0.3 mL Met (130 mM), 0.3 mL NBT (0.75 mM), 0.3 mL EDTA-Na_2_ (0.1 mM), 0.3 mL riboflavin (20 µM), 0.1 mL enzyme extract, and 0.5 mL distilled water in a total volume of 3.3 mL. The mixtures were illuminated by light (4000 Lx) for 20 min at 28 °C, and the absorbance was then determined at 560 nm (Shimadzu UV-2600, Tokyo, Japan). One unit of SOD activity was defined as the amount of enzyme that would inhibit 50% of NBT photoreduction, and expressed as U min^−1^·g^−1^.

POD activity was based on the measurement of guaiacol oxidation at 470 nm in the presence of H_2_O_2_. The collected supernatant (100 μL) was mixed with 3.0 mL of 25 mM guaiacol and 200 μL of 50 mM H_2_O_2_. Oxidation of guaiacol was determined at 470 nm for 3 min. One unit of POD activity was defined as an increment of 0.01 in absorbance per minute at 470 nm (Shimadzu UV-2600), and expressed as U min^−1^ g^−1^.

PAL activity was determined by using a PAL assay kit (Beijing Leagene Biotechnology Co., Ltd, China) monitoring the absorbance of the tested sample at 290 nm using an M5 microplate reader (Molecular Devices Corporation, Sunnyvale, CA, USA) and expressed as U·h^−1^·g^−1^.

PPO activity was based on the measurement of catechol oxidation at 420 nm. The collected supernatant (200 μL) was mixed with 4.0 mL of 50 mM acetate buffer (pH 5.5) and 1.0 mL of 50 mM catechol. Oxidation of catechol was determined at 420 nm for 5 min. One unit of PPO activity was defined as an increment of 0.01 in absorbance per hour at 420 nm (Shimadzu UV-2600), and expressed as U·h^−1^·g^−1^.

### 2.8. Statistical Analysis

All data calculated from three physical and chemical experiments was expressed as the mean with standard error (SE). The SPSS software (Version 17.0, SPSS Inc., Chicago, IL, USA) was applied to determine the mean differences using Duncan’s multiple range test at *P* < 0.01 and *P* < 0.05, respectively.

## 3. Results and Discussion

### 3.1. Antifungal Efficacy of FFE on In Vivo Mycelial Growth of P. italicum

**Figure 1:**
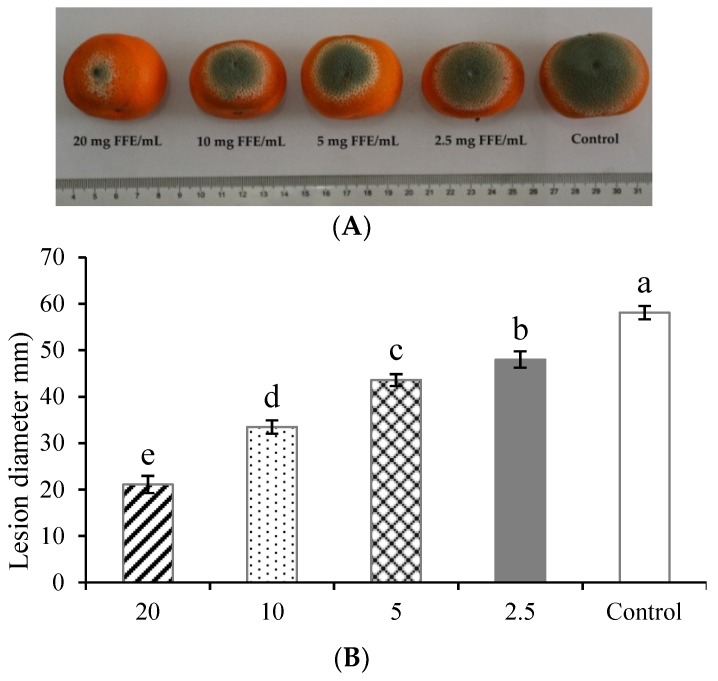

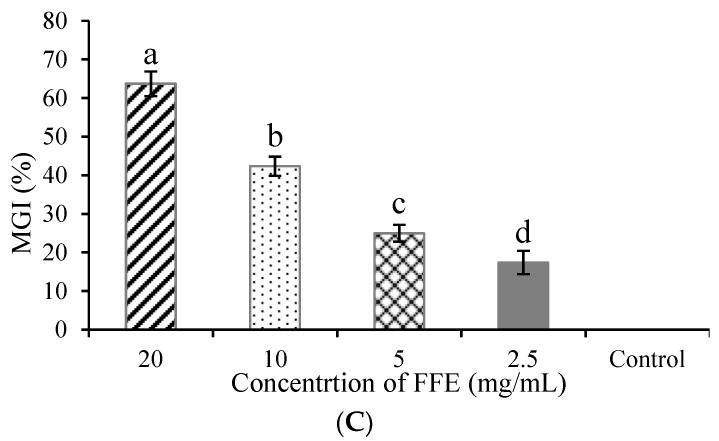
Antifungal efficacy of Ficus hirta Vahl. fruits extract (FFE) on *in vivo* mycelial growth of *Penicillium italicum* in Xinyu tangerines. Disease development (**A**), lesion diameter (**B**), and MGI (**C**) were measured after 7 days of incubation at 25 °C. Bars indicate the mean of fifteen fruits ± standard deviation (SD) and means labeled with different letters were significantly different according to Duncan’s multiple range test at *P* < 0.01.

In the present study, the *in vivo* antifungal efficacy of postharvest blue mold in Xinyu tangerines inoculated with *P. italicum* were significantly reduced by FFE treatment at various concentrations. As shown in Figure 1A,B, the disease development and lesion diameters in FFE-treated Xinyu tangerines were much lower than in control fruits over 7 days of incubation at 25 °C (*P* < 0.01). Concurrently, the MGI of blue mold in FFE-treated (20, 10, 5, and 2.5 mg/mL) Xinyu tangerines were 63.68%, 42.34%, 24.96%, and 17.38%, which indicates that FFE possessed strong antifungal efficacy and efficiently inhibited *in vivo* mycelial growth of *P. italicum* in a dose-dependent manner (Figure 1C). In addition, the EC_50_ value for FFE causing 50% inhibition of mycelial growth of *P. italicum* in Xinyu tangerines was 12.543 mg/mL.

Previously, our studies have demonstrated that FFE*,* a plant-derived potential fungicide, had a broad antifungal spectrum and significantly inhibited growth of fungal pathogens in citrus, kiwifruits, pears, and eggplants [16]. In particular, pinocembroside, a flavonone compound isolated from the ethanol extract of *F. hirta* Vahl. Fruits, was responsible for antifungal activity against *P. italicum* [18,19]. In the present *in vivo* test, disease development in Xinyu tangerines caused by *P. italicum* was significantly reduced with increasing concentrations of FFE treatment (*P* < 0.01). In addition, the FFE–CS coating effectively reduced natural infection and prolonged storage life of Xinyu tangerines during storage at 5 °C for 90 days. These results were in agreement with previous reports that application of plant extracts could reduce *Penicillium* decay of citrus fruit [22,23,24,25]. Therefore, FFE–CS was used as an edible coating for fresh Xinyu tangerines.

### 3.2. Effect of FFE-CS Coating on Postharvest Fruit Quality of Xinyu Tangerines

**Figure 2:**
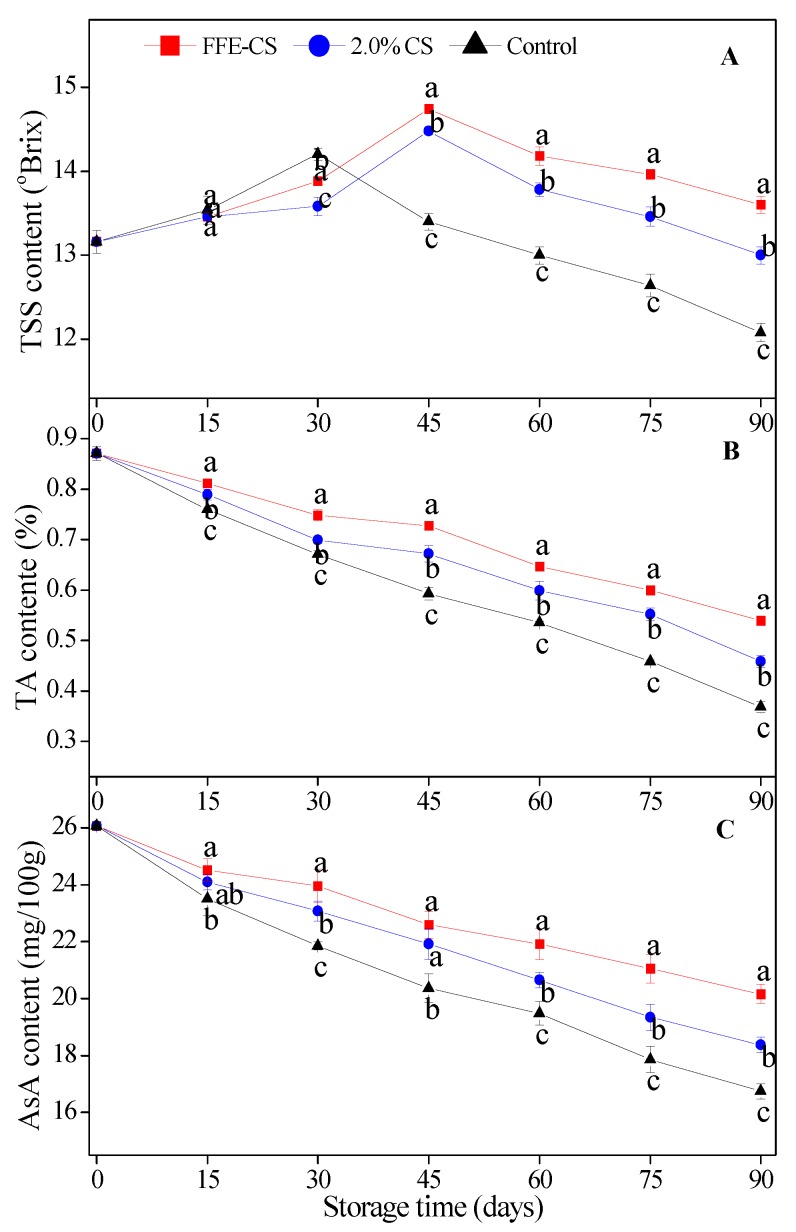
Effect of FFE-CS coating on the contents of total soluble solid (TSS) (**A**), titrable acid (TA) (**B**) and ascorbic acid (AsA) (**C**) of Xinyu tangerines during cold storage of 90 days. Differences between treatments for each time were analyzed using Duncan’s multiple range test at *P* < 0.05.

TSS in citrus juice is made up of sugars, acids, soluble pectins, vitamins, and some soluble proteins [26]. TSS content in coated or control fruits firstly increased, owing to the postharvest ripening, then decreased slightly in the later storage period. However, the peak time of TSS contents was different for different treatments (e.g., 45 days for FFE–CS and 2.0% CS coatings, and 30 days for control (Figure 2A). Furthermore, the peak value of TSS content in FFE–CS-coated fruits was significantly higher than that in 2.0% CS-coated and control fruits (*P* < 0.05). Therefore, the TSS content in FFE–CS-coated fruits were generally higher than 2.0% CS-coated and control in the later period of storage (45 days to 90 days). Similar results were reported by Chien and Chou [27] and Tao et al. [28], for Tankan fruit and Satsuma mandarins coated with various concentrations of chitosan and sucrose-based polymers (SBP). These results are highly consistent with the report of Chen et al. [2], finding that the clove essential oil (5.0 g·L^−1^) incorporated into 1.0% CMC-based edible coating had a striking efficiency in prevention of the loss of TSS. Therefore, the 2.0% CS edible coating with FFE formed a semi-permeable coating around Xinyu tangerines which inhibited their respiration and nutrient consumption, and maintained the high level of TSS.

The main components of TA are organic acids which participate in the respiration of the plant, and the TA content is regarded as an important indicator for evaluation of the respiration rate of horticultural crops [29]. TA content gradually reduced in all treatments as storage time increased, but the FFE–CS coating significantly retarded the rate of descension of TA (*P* < 0.05). At the end of storage (90 days), TA content with FFE–CS coating was 0.539%, while it was 0.468% and 0.357% in 2.0% CS coating and control groups, respectively. The high reduction in TA content of FFE–CS coating could have contributed to the slowing of the respiration rate, thereby delaying the degradation of TA [10,17,30,31,32,33].

AsA is regarded as one of key determinants in evaluation of citrus fruit quality. As illustrated in Figure 2C, Xinyu tangerines in the control group showed a rapid decrease in AsA content, and this decreasing trend continued to the end of cold storage. With the value decreasing 35.49%, the overall AsA content was significantly lower than the in fruits with the other two coatings (*P* < 0.05). The AsA content of FFE–CS-coated fruits was significantly higher than those with 2.0% CS coating and controls. These results are highly consistent with those of Chen et al. [10] and Gao et al. [32], where a slow decline in AsA was recorded in citrus fruits treated with 1.5% chitosan coating enriched with FFE and cinnamaldehyde. Thus, our results verified that FFE–CS coating caused prominent inhibition of the degradation and consumption of AsA in Xinyu tangerines. The high reduction in AsA content of FFE–CS coating could be responsible for delaying the senescence and prolonging storage life of coated Xinyu tangerines, and, as will be discussed later, MDA content increased during storage.

### 3.3. Effect of FFE-CS Coating on Decay Rate and Weight Loss of Xinyu Tangerines

**Figure 3:**
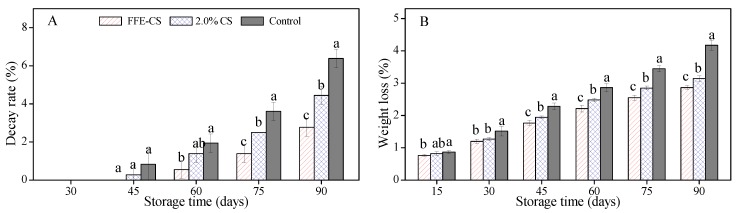
Effect of FFE-CS coating on decay rate (**A**) and weight loss (**B**) of Xinyu tangerines during cold storage of 90 days. Differences between treatments for each time were analyzed using Duncan’s multiple range test at *P* < 0.05.

The percentages of decay rate and weight loss are two important indicators for evaluating storability of harvested citrus fruit [10,32]. As shown in Figure 3A, during the first 30 days of cold storage, the decay rate in coated and control groups was almost zero. The rotten fruit appeared in control, 2.0% CS-, and FFE–CS-coated groups at 45 days and 60 days, respectively. The decay rate in coated fruits was much lower than that in the control group: 2.78% for FFE–CS, 4.44% for 2% CS, and 6.39% for control, respectively, at the end of cold storage, and a significant difference was observed between Xinyu tangerines coated with FFE–CS and with 2.0% CS. There are numerous previous studies reporting the effect of plant extracts incorporated into CS coating on reducing the pathogenic decay of horticultural crops [5,10,34,35,36]; however, the *in vivo* ability of FFE to inhibit fungal growth and the fresh preservation effect of FFE–incorporated chitosan coating to reduce decay rate have not yet been investigated for Xinyu tangerines. According to Chen et al. [16], FFE from hairy fig (*Ficus hirta* Vahl.) fruits exhibited a variety of biological activities and inhibited the fungal growth of pathogens (*Alternaria citri*, *Botryosphaeria dothidea*, *Botrytis cinereal*, *Geotrichum citri-aurantii*, *Penicillium digitatum*, *P. italicum*, etc.). In line with our findings, FFE may be considered as a safe natural preservative for reducing fungal decay in horticultural crops.

Weight losses in coated and control groups increased with storage time as presented in Figure 3B, which was mainly due to the water loss caused by transpiration. Weight loss in control fruits reached values of 4.17 ± 0.15% at the end of storage, while these were 3.14 ± 0.08% and 2.86 ± 0.06% in 2.0% CS- and FFE–CS-coated groups, respectively, with a significant difference between treatments (*P* < 0.05). It is apparent that FFE–CS coating showed the lowest values during the whole cold storage. The weight loss in FFE–CS-coated fruits declined by 31.41% at 90 days compared with that of the control. Several previous reports have confirmed that coatings of almond carboxymethyl cellulose (CMC) [2,9,29,37,38,39], chitosan [5,9,10,32], hydroxypropyl methylcellulose (HPMC) [8,9,40,41], alginate [17,42], and sucrose-based polymers [9,28] could reduce weight loss of citrus fruit. In our study, FFE–CS coating exhibited approximately less weight loss than the 2.0% CS-coated and control groups. This was likely associated with the FFE–CS coating around Xinyu tangerines providing a semi-permeable barrier to moisture and respiration, therefore reducing the loss of water and nutrients (sugar, organic acid, etc.). Overall, the incorporation of FFE into 2.0% CS edible coatings to delay water loss and enhance nutrients retention is desirable.

### 3.4. Effect of FFE-CS Coating on Respiration Rate of Xinyu tangerines

**Figure 4:**
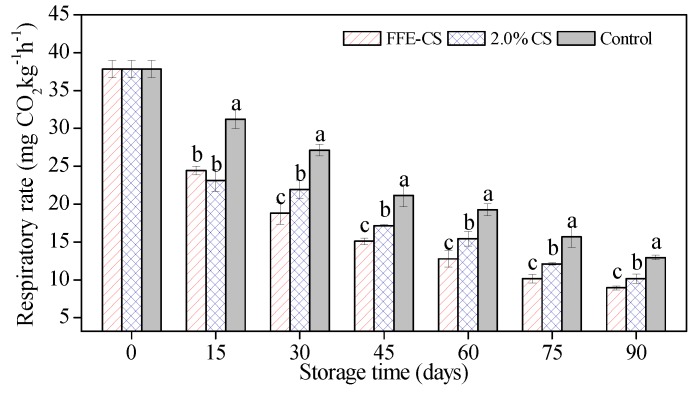
Effect of FFE–CS coating on respiration rate of Xinyu tangerines during cold storage for 90 days. Differences between treatments for each time were analyzed using Duncan’s multiple range test at *P* < 0.05.

The respiration rate of Xinyu tangerines was 37.82 ± 1.14 mg·kg^−1^·h^−1^ at the beginning of the storage. It was observed in Figure 4 that the respiration rate of the control group gradually decreased as the storage time increased and always had significantly higher values compared to those of coated groups. Moreover, there was a significant difference (*P* < 0.05) between two coated groups of FFE–CS and 2.0% CS after 15 days of storage. Coated Xinyu tangerines generally had lower respiration rates than control fruits (Figure 4), likely due to the modification of internal gas by FFE–CS and 2.0% CS coatings [7,30]. Similar findings have been reported by Arnon et al. [43], Chen et al. [2,39], Togrul and Arslan [37], and Zeng et al. [38] in citrus fruit coated with carboxymethyl cellulose (CMC)-based edible coatings. In this experiment, the addition of FFE into 2.0% CS resulted in a decrease in the respiration rates of coated Xinyu tangerines (thereby inhibiting the respiration of fruits and pathogens) such that fruits coated with 2.0% CS containing FFE had consistently lower respiration rates than 2.0% CS-coated and control fruits. Similarly, the respiration rate of Newhall oranges and Ponkan mandarins coated with chitosan edible coatings containing FFE and cinnamaldehyde has been shown to decrease compared to untreated fruit [10,32].

### 3.5. Effect of FFE–CS Coating on MDA Content of Xinyu Tangerines

**Figure 5:**
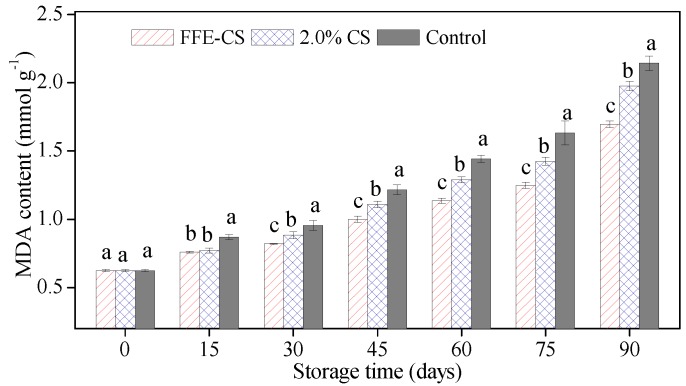
Effect of FFE–CS coating on malondialdehyde (MDA) content of Xinyu tangerines during cold storage of 90 days. Differences between treatments for each time were analyzed using Duncan’s multiple range test at *P* < 0.05.

MDA is the final product of lipid peroxidation, related to senescence, and is used as one of the direct indices of cell oxidative damage. As shown in Figure 5, the uncoated Xinyu tangerines (control) showed a rapid increase in MDA content, and this increasing trend continued to the end of cold storage, with the value increasing 2.45 times. The overall MDA content in the control group was significantly (*P* < 0.05) higher than that in the two coated groups. Xinyu tangerines coated with FFE–CS showed a lower MDA content compared to 2.0% CS coating and control. These results are highly consistent with those of Chen et al. [10] and Shah et al. [31], where a slow rise in MDA was recorded in citrus fruits treated with 1.5% chitosan coating enriched with FFE, and CMC edible coating containing silver nanoparticles. Therefore, our work has confirmed that FFE–CS is a promising treatment for inhibition of the MDA accumulation of Xinyu tangerines. This inhibition of MDA content could be attributed to a delay in the senescence of FFE–CS-coated fruits and to the retention of AsA of coated fruits. To further understand this changing mechanism, the protective enzyme activities that postpone lipid peroxidation and cell aging should be determined in future analysis. 

### 3.6. Effect of FFE–CS Coating on Protective Enzyme Activities of Xinyu Tangerines

**Figure 6:**
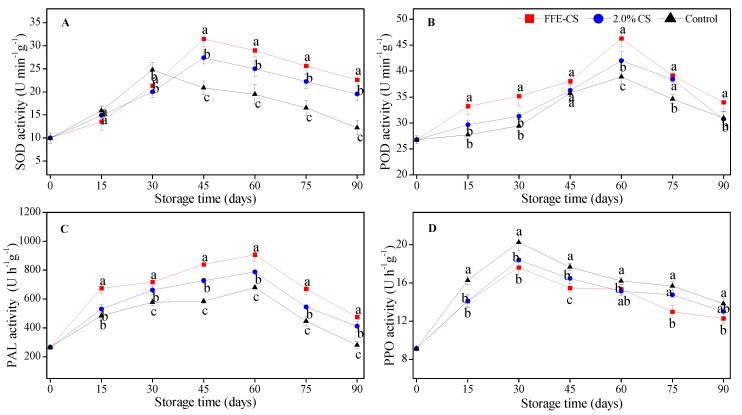
Effect of FFE–CS coating on the activities of superoxide dismutase (SOD) (**A**), peroxidase (POD) (**B**), polyphenol oxidase (PPO) (**C**), and phenylalanine ammonia-lyase (PAL) (**D**) of Xinyu tangerines during cold storage of 90 days. Differences between treatments for each time were analyzed using Duncan’s multiple range test at *P* < 0.05.

The activities of SOD, POD, PAL, and PPO are closely related to the resistance of oxidation and disease in plant tissue [44]. As displayed in Figure 6A, SOD activity in control fruits raised to reach its peak at 30 days of storage, and dropped rapidly to by the end of storage, while the SOD activity in FFE–CS- and 2.0% CS-coated groups exhibited a quick increase on the 45th day of storage, and then decreased gradually for the following day. After 30 days, the SOD activity in FFE–CS-coated fruits was significantly higher than in 2.0% CS-coated and control fruits (*P* < 0.05). Therefore, the SOD activity in FFE–CS-coated fruits was generally higher than 2.0% CS-coated and control in the later stage of storage period (45 days to 90 days). 

POD activity in the coated and control fruits increased dramatically to reach its peak at 60 days of storage and then declined (Figure 6B). The peak value of POD activity in FFE–CS-coated fruits at 60 days was 46.26 ± 1.52 U·min^−1^·g^−1^, significantly (*P* < 0.05) higher than that in 2.0% CS-coated and control fruits. It was confusing that the overall POD activity in FFE–CS-coated fruits was significantly (*P* < 0.05) higher than that of the control fruits, while the MDA accumulation was inhibited with significant differences compared to 2.0% CS-coated and control groups (Figure 5).

As shown in Figure 6C, PAL activity in FFE–CS- and 2.0% CS-coated fruits increased sharply in the early stage of the storage period and reached peak values of 906.5 ± 49.6 U·h^−1^·g^−1^ and 787.5 ± 10.5 U·h^−1^·g^−1^, respectively, at 60 days of storage, which shown comparatively equals 1.34 times and 1.16 times higher than that in control fruits, and then decreased. It was obvious that the overall PAL activity in FFE–CS-coated fruits was significantly (*P* < 0.05) higher than that of the 2.0% CS-coated and control fruits. 

PPO activity in the coated and control fruits followed a clear tendency of increasing dramatically to reach their peaks at 30 days of storage and then decreasing as storage time increased (Figure 6D). The peak value of PPO activity in the control fruits was much higher than that in the coated fruits: 20.26 ± 0.82 U·h^−1^·g^−1^ for control, 18.40 ± 0.21 U·h^−1^·g^−1^ for 2% CS, and 17.64 ± 0.76 U·h^−1^·g^−1^ for FFE–CS at 30 days of cold storage. It is apparent that FFE–CS coating showed the minimum values of PPO activity during the whole cold storage. 

Fruit senescence leads to the overproduction and accumulation of reactive oxygen species (ROS); this is considered the most important metabolic process in plants that causes damage to cell membranes and reduces the storability of fruits [45]. High activity of antioxidant and defense-related enzymes can effectively reduce the accumulation of ROS and MDA and alleviate oxidative damage, and thereby delay fruit senescence and prolonged storage life. SOD and POD are two important antioxidant enzymes which prevent lipid peroxidation of membranes caused by an excess of ROS [44,46]. PAL, as the key enzyme in the phenylpropanoid pathway, regulates the synthesis of phenolics, phytoalexins, and lignin, which are directly involved in defense responses by preventing pathogens from infecting the host fruits [44]. The resulting phenolics are oxidized by PPO in the presence of ROS, typically resulting in tissue browning and nutritional quality decline associated with increased PPO activity [47]. In this present study, our results demonstrated that FFE–CS coating induced increases in the activities of SOD, POD, and PAL, and decreases in the activity of PPO and the accumulation of MDA (Figure 5 and Figure 6). These findings were supported by the reports of Chen and co-authors [17] who found that Nanfeng mandarins coated with an alginate-based edible coating containing FFE increased antioxidant and defense-related enzymes. In addition to this, similar results have been reported by Chen et al. [10] when Newhall navel oranges were coated with a 1.5% CS coating containing FFE, Duan et al. [48] when Ponkan mandarins were coated with a wax coating (SP-1) incorporating cinnamaldehyde, and Adiletta et al. [33] when loquat fruits were coated with a 1.0% chitosan coating. Thus, our results may imply that these antioxidant and defense-related enzymes be collectively induced by FFE–CS coating to enhance disease resistance and prolong storage life for Xinyu tangerine preservation.

## 4. Conclusions

In this study, treatments with FFE at different concentrations demonstrated its *in vivo* antifungal efficacy for controlling blue mold in citrus fruit caused by *P. italicum*. Our work also showed the benefit of FFE–CS coating to delay the fruit senescence process, improve postharvest quality, and prolong storage life of Xinyu tangerines. This coating helped to maintain the lower reduction of TSS, TA, and AsA content by depressing the respiration rate. In addition, the FFE–CS coating significantly increased SOD, POD and PAL activities and decreased PPO activity and MDA content, thus reducing oxidative stress and delaying lipid peroxidation to membranes. Xinyu tangerines subjected to the FFE–CS coating showed slower rates of fruit decay and weight loss, which corresponded to improved storability and prolonged storage life. According to the results from this study, FFE–CS coating treatment showed the best preservation effect for harvested Xinyu tangerines. 

## References

[1] Cai N., Chen J.Y., Peng X., Chen C.Y. (2018). Application of principle component analysis on postharvest characteristic Xinyu tangerines during ambient temperature storage. China Fruits.

[2] Chen C.Y., Zheng J.P., Wan C.P., Chen M., Chen J.Y. (2016). Effect of carboxymethyl cellulose coating enriched with clove oil on postharvest quality of Xinyu mandarin oranges. Fruits.

[3] Chen C.Y., Wan C.P., Jian H.Z., Zou Z.Q., Zeng T., Chen J.Y. (2019). Effects of different cold storage temperature on postharvest quality of Xinyu tangerines. Mol. Plant Breed..

[4] Chen C.Y., Fu Y., Wan C.P., Chen J.Y. (2019). Principal component analysis on influence of hot water dipping on postharvest storage quality of Xinyu tangerines during cold storage. Food Ferm. Ind..

[5] Peng X., Wan C.P., Chen C.Y., Chen J.Y. (2017). Effects of the complex coating of chitosan with extract of Cynanchum atratum on cold storage of navel orange. J. Hunan Agric. Univ..

[6] Falguera V., Quintero J.P., Jiménez A., Muñoz J.A., Ibarz A. (2011). Edible films and coatings: Structures, active functions and trends in their use. Trends Food Sci. Technol..

[7] Grande-Tovar C.D., Chaves-Lopez C., Serio A., Rossi C., Paparella A. (2018). Chitosan coatings enriched with essential oils: Effects on fungi involve in fruit decay and mechanisms of action. Trends Food Sci. Technol..

[8] Valencia-Chamorro S.A., Palou L., del Río M.Á., Pérez-Gago M.B. (2011). Performance of hydroxypropyl methylcellulose (HPMC)-lipid edible coatings with antifungal food additives during cold storage of ‘Clemenules’ mandarins. LWT Food Sci. Technol..

[9] Palou L., Valencia-Chamorro S.A., Pérez-Gago M.B. (2015). Antifungal Edible Coatings for Fresh Citrus Fruit: A Review. Coatings.

[10] Chen C.Y., Cai N., Chen J.Y., Peng X., Wan C.P. (2018). Chitosan-Based Coating Enriched with Hairy Fig (Ficus hirta Vahl.) Fruit Extract for “Newhall” Navel Orange Preservation. Coatings.

[11] Yi T., Chen Q.L., He X.C., So S.W., Lo Y.L., Fan L.L., Xu J., Tang Y., Zhang J.Y., Zhao Z.Z., Chen H.B. (2013). Chemical quantification and antioxidant assay of four active components in *Ficus hirta* root using UPLC-PAD-MS fingerprinting combined with cluster analysis. Chem. Cent. J..

[12] Cheng J., Yi X.M., Chen H.Y., Wang Y.H., He X.J. (2017). Anti-inflammatory phenylpropanoids and phenolics from *Ficus hirta* Vahl. Fitoterapia.

[13] Ya J., Zhang X.Q., Wang Y., Zhang Q.W., Chen J.X., Ye W.C. (2010). Two new phenolic compounds from the roots of *Ficus hirta*. Nat. Prod. Res..

[14] Zeng Y.W., Liu X.Z., Lv Z.C., Peng Y.H. (2012). Effects of *Ficus hirta* Vahl. (Wuzhimaotao) extracts on growth inhibition of HeLa cells. Exp. Toxicol. Pathol..

[15] Chen Q., Ye S.X. (2012). Antibacterial activity of *Ficus hirta* Vahl. by chromotest microassay. J. Anhui Agric. Sci..

[16] Chen C.Y., Wan C.P., Peng X., Chen Y.H., Chen M., Chen J.Y. (2015). Optimization of Antifungal Extracts from *Ficus hirta* Fruits Using Response Surface Methodology and Antifungal Activity Tests. Molecules.

[17] Chen C.Y., Peng X., Zeng R., Chen M., Wan C.P., Chen J.Y. (2016). *Ficus hirta* fruits extract incorporated into an alginate-based edible coating for Nanfeng mandarin preservation. Sci. Hortic..

[18] Wan C.P., Han J.X., Chen C.Y., Yao L.L., Chen J.Y., Yuan T. (2016). Monosubstituted Benzene Derivatives from Fruits of *Ficus hirta* and Their Antifungal Activity against Phytopathogen *Penicillium italicum*. J. Agric. Food Chem..

[19] Wan C.P., Chen C.Y., Li M.X., Yang Y.X., Chen M., Chen J.Y. (2017). Chemical Constituents and Antifungal Activity of *Ficus hirta* Vahl. Fruits. Plants.

[20] Hodges D.M., DeLong J.M., Forney C.F., Prange R.K. (1999). Improving the thiobarbituric acid-reactive-substances assay for estimating lipid peroxidation in plant tissues containing anthocyanin and other interfering compounds. Planta.

[21] Sala J.M., Lafuente M.A.T. (2000). Catalase enzyme activity is related to tolerance of mandarin fruits to chilling. Postharvest Biol. Technol..

[22] Jafari S., Hassandokht M., Javan-Nikkhah M. (2014). Effects of dog rose and watercress extracts on control of green mould decay and postharvest quality of orange fruits. Nat. Prod. Res..

[23] Tayel A.A., Moussa S.H., Salem M.F., Mazrou K.E., El-Tras W.F. (2015). Control of citrus molds using bioactive coatings incorporated with fungal chitosan/plant extracts composite. J. Sci. Food Agric..

[24] Mekbib S.B., Regnier T.J., Korsten L. (2007). Control of *Penicillium digitatum* on citrus fruit using two plant extracts and study of their mode of action. Phytoparasitica.

[25] Sanzani S.M., Schena L., Ippolito A. (2014). Effectiveness of Phenolic Compounds against Citrus Green Mould. Molecules.

[26] Teerachaichayut S., Ho H.T. (2017). Non-destructive prediction of total soluble solids, titratable acidity and maturity index of limes by near infrared hyperspectral imaging. Postharvest Biol. Technol..

[27] Chien P.J., Chou C.C. (2006). Antifungal activity of chitosan and its application to control post-harvest quality and fungal rotting of Tankan citrus fruit (*Citrus tankan* Hayata). J. Sci. Food Agric..

[28] Tao N.G., Ao T.T., Liu Y.J., Huang S.R. (2012). Effect of sucrose-based polymers on quality of Satsuma mandarin fruit (*Citrus unshiu* Marc. cv. Miyagawa Wase). Int. J. Food Sci. Technol..

[29] Chen M., Xie X.L., Lin Q., Chen J.Y., Grierson D., Yin X.R., Sun C.D., Chen K.S. (2013). Differential expression of organic acid degradation-related genes during fruit development of Navel oranges (*Citrus sinensis*) in two habitats. Plant Mol. Biol. Rep..

[30] Xing Y., Xu Q., Yang S., Chen C., Tang Y., Sun S., Zhang L., Che Z., Li X. (2016). Preservation Mechanism of Chitosan-Based Coating with Cinnamon Oil for Fruits Storage Based on Sensor Data. Sensors.

[31] Shah S.W.A., Jahangir M., Qaisar M., Khan S.A., Mahmood T., Saeed M., Farid A., Liaquat M. (2015). Storage Stability of Kinnow Fruit (*Citrus reticulata*) as Affected by CMC and Guar Gum-Based Silver Nanoparticle Coatings. Molecules.

[32] Gao Y., Kan C.N., Chen M., Chen C.Y., Chen Y.H., Fu Y.Q., Wan C.P., Chen J.Y. (2018). Effects of Chitosan-Based Coatings Enriched with Cinnamaldehyde on Mandarin Fruit cv. Ponkan during Room-Temperature Storage. Coatings.

[33] Adiletta G., Pasquariello M., Zampella L., Mastrobuoni F., Scortichini M., Petriccione M. (2018). Chitosan Coating: A Postharvest Treatment to Delay Oxidative Stress in Loquat Fruits during Cold Storage. Agronomy.

[34] Won J.S., Lee S.J., Park H.H., Song K.B., Min S.C. (2018). Edible Coating Using a Chitosan-Based Colloid Incorporating Grapefruit Seed Extract for Cherry Tomato Safety and Preservation. J. Food Sci..

[35] Kanetis L., Exarchou V., Charalambous Z., Goulas V. (2017). Edible coating composed of chitosan and Salvia fruticosa Mill. extract for the control of gray mold of table grapes. J. Sci. Food Agric..

[36] Moradi M., Tajik H., Razavi Rohani S.M., Oromiehie A.R., Malekinejad H., Aliakbarlu J., Hadian M. (2012). Characterization of antioxidant chitosan film incorporated with *Zataria multiflora* Boiss essential oil and grape seed extract. LWT Food Sci. Technol..

[37] Togrul H., Arslan N. (2004). Carboxymethyl cellulose from sugar beet pulp cellulose as a hydrophilic polymer in coating of mandarin. J. Food Eng..

[38] Zeng R., Zhang A., Chen J., Fu Y. (2013). Impact of carboxymethyl cellulose coating enriched with extract of *Impatiens balsamina* stems on preservation of ‘Newhall’ navel orange. Sci. Hortic..

[39] Chen C.Y., Peng X., Zeng R., Wan C.P., Chen M., Chen J.Y. (2017). Physiological and biochemical responses in cold-stored citrus fruits to carboxymethyl cellulose coating containing ethanol extract of *Impatiens balsamina* L. stems. J. Food Process. Pres..

[40] Valencia-Chamorro S.A., Pérez-Gago M.B., del Río M.Á., Palou L. (2009). Effect of antifungal hydroxypropyl methylcellulose (HPMC)–lipid edible composite coatings on postharvest decay development and quality attributes of cold-stored ‘Valencia’ oranges. Postharvest Biol. Technol..

[41] Contreras-Oliva A., Rojas-Argudo C., Pérez-Gago M.B. (2012). Effect of solid content and composition of hydroxypropyl methylcellulose-lipid edible coatings on physico-chemical and nutritional quality of ‘Oronules’ mandarins. J. Sci. Food Agric..

[42] Aloui H., Khwaldia K., Sanchez-Gonzalez L., Muneret L., Jeandel C., Hamdi M., Desobry S. (2014). Alginate coatings containing grapefruit essential oil or grapefruit seed extract for grapes preservation. Int. J. Food Sci. Technol..

[43] Arnon H., Zaitsev Y., Porat R., Poverenov E. (2014). Effects of carboxymethyl cellulose and chitosan bilayer edible coating on postharvest quality of citrus fruit. Postharvest Biol. Technol..

[44] Ballester A.R., Lafuente M.T., González-Candelas L. (2006). Spatial study of antioxidant enzymes, peroxidase and phenylalanine ammonia-lyase in the citrus fruit–*Penicillium digitatum* interaction. Postharvest Biol. Technol..

[45] Gill S.S., Tuteja N. (2010). Reactive oxygen species and antioxidant machinery in abiotic stress tolerance in crop plants. Plant Physiol. Biochem..

[46] Mittler R. (2002). Oxidative stress, antioxidants and stress tolerance. Trends Plant Sci..

[47] Yoruk R., Marshall M.R. (2003). Physicochemical properties and function of plant polyphenol oxidase: A review. J. Food Biochem..

[48] Duan X., OuYang Q., Tao N. (2018). Effect of applying cinnamaldehyde incorporated in wax on green mould decay in citrus fruits. J. Sci. Food Agric..

